# Infant Motor Competence Predicts Executive Functions in Preschoolers: The Role of Sleep

**DOI:** 10.3390/bs16020288

**Published:** 2026-02-17

**Authors:** Chao Liu, Yuzhu Zhang, Xi Liang

**Affiliations:** 1 Research Center for Child Development, Beijing Key Laboratory of Learning and Cognition, School of Psychology, Capital Normal University, Beijing 100048, China; 2 Tianjin Fulun Middle School, Tianjin 300142, China

**Keywords:** motor skills, cognitive ability, executive functions, sleep, moderated mediation

## Abstract

The theory of embodied cognition suggests that cognitive development in infancy relies on sensorimotor experiences gained through interaction with the environment. Additionally, the need for more sleep in early childhood may be linked to the development of executive functions. This study examined 255 children in Beijing to investigate these ideas. Motor skills were evaluated at 6 months and 1 year old, cognitive abilities at 2 years old, and executive functions at 3 years old. The results showed that strong motor skills in infancy predicted better executive functions in preschool, and this relationship was fully mediated by cognitive abilities. Furthermore, the mediating effects of cognitive abilities on executive functions were stronger in preschoolers with more and better sleep. In conclusion, motor competence and sufficient sleep are crucial for the development of executive function.

## 1. Introduction

Executive functions (EFs) are a set of higher-order cognitive processes such as working memory, response inhibition, and mental flexibility necessary for control over behavior (Diamond, 2013; Miyake et al., 2000). Individual differences in EFs during early childhood predict academic achievement and mental health (Morgan et al., 2019; Ren & Hu, 2019). Motor skills and physical activity are stronger predictors of high executive functioning (Gandotra et al., 2022; Song et al., 2023), although not among all study populations and in all contexts, suggesting the importance of additional factors. One potential factor influencing EFs development either independently or by influencing the effects of motor competence is sleep, given the known importance of sleep in other cognitive processes such as memory consolidation. Indeed, several recent studies have examined the contributions of sleep quantity and quality to early EFs development (Bernier et al., 2021; Mäkelä et al., 2020; Morales-Muñoz et al., 2021; Nieto et al., 2022; Sun et al., 2022; Woods et al., 2023), but have not considered potential interactions between sleep and motor competence. Therefore, the present study investigated motor competence during infancy and sleep quality as predictors of EFs development at 3 years.

### 1.1. Infant Motor Competence and Later Cognitive Development

Motor competence is the ability to perform a range of motor tasks, such as locomotion and object manipulation (Cattuzzo et al., 2016). According to Piaget’s theory, the first concepts and knowledge acquired in infancy are based on sensorimotor experiences, implying that a child’s ability to move has important implications for the emergence of cognitive abilities (Piaget, 1936). Consistent with this idea, more recent theoretical accounts posit that the body’s interactions with the world are essential for cognitive development (Wilson, 2002).

Motor competence is often broadly divided into gross and fine motor skills. Gross motor skills include balance and various limb and trunk movements for independent sitting, crawling, walking, running, climbing, and manipulating large objects (Davis et al., 2000). For infants, learning these gross motor skills can fundamentally alter their interactions with the world and with others (Adolph & Hoch, 2020; Campos et al., 2000). Independent sitting allows infants to explore objects more freely, as their hands are no longer needed for support. It also provides the child with a wider visual field, enhancing access to perceptual information (Marcinowski et al., 2019). In fact, gross motor skills are positively associated with cognitive development in toddlers (Veldman et al., 2019). Moreover, standing and walking are the most important milestones in infancy related to adult intelligence (Flensborg-Madsen & Mortensen, 2018).

Fine motor skills allow intricate precise movements using small muscle groups, especially of the hands, and require high levels of hand–eye coordination (Luo et al., 2007). According to the nimble-hands, nimble-minds hypotheses (Suggate & Stoeger, 2017), a child with advanced fine motor skills will engage in richer sensorimotor interactions with objects, and will show a greater understanding of terms referring to objects that are easily manipulable. Indeed, a growing body of evidence has supported this prediction (Martzog & Suggate, 2019; Zuccarini et al., 2017). Therefore, it is believed that development of both gross and fine motor competence can facilitate an infant’s engagement with their environment, which in turn provides opportunities for both procedural and declarative knowledge acquisition.

### 1.2. Infant Motor Competence and Executive Functions in Preschool Years

Given these associations, it is possible that EFs and motor competence share common neural mechanisms. EFs evolved through the need to control motor behaviors (Koziol et al., 2012), and related neuroplastic processes in the frontal lobe mediate the development of both self-guided locomotion and EFs (Koziol & Lutz, 2013). Ito (1993) also suggested contributions from the cerebellum, as circuits within this structure control both movement and thoughts related to movement. Thus, the control and manipulation of a body part is similar to the control and manipulation of thoughts. Further, a recent functional near-infrared spectroscopy study revealed that both active locomotion and EFs engage the prefrontal cortex during early development (Weibley et al., 2021).

Cross-sectional studies of preschoolers also support potential links between motor competence and EFs, although the strength of the association depends on motor task difficulty (Cook et al., 2019; Gandotra et al., 2022; Maurer & Roebers, 2019; Veer et al., 2020). Recent theoretical accounts have offered explanations for this variable relationship based on reciprocity and automaticity (Kim et al., 2018). There is reciprocity when motor competence and EFs develop and improve together (McClelland & Cameron, 2019), while automaticity refers to the competition between motor and cognitive tasks for attentional resources (Floyer-Lea & Matthews, 2004). When a new motor skill becomes automatic, attention can be devoted to more complex cognitive tasks (Cameron et al., 2012). Conversely, infants and toddlers lacking such automaticity may have to devote more attention to motor performance, placing a constraint on cognitive development and learning.

Validating this association between motor competence and EFs development requires longitudinal studies of large study cohorts. Our previous study found that general cognitive abilities mediate the relation between motor competence and EFs (Wu et al., 2017), supporting infant motor competence as an important predictor of later EFs, but cognitive flexibility was not included and mediation was not tested over time.

### 1.3. Sleep as a Moderator

Sleep is critical for brain and cognitive development (Mason et al., 2021), and the maturation of sleep is one of the most crucial developmental processes during early childhood (Voltaire & Teti, 2018). Unfortunately, disruptions in the quantity and quality of sleep occur in up to 33% of kindergarteners, with an even higher prevalence in Asian countries, especially China (Hua et al., 2022; Liu et al., 2012). Children’s sleep problems are associated with deficits in higher-order cognitive functions and dysregulation (Astill et al., 2012; Williams et al., 2023). Meta-analytic findings among school-age children indicate that lower sleepiness, higher sleep quality, and longer sleep duration are consistently related to better school functioning (Dewald et al., 2010). Similar patterns have been observed in preschool-aged children, with sleep-related difficulties associated with poorer executive functioning and predicting lower neurobehavioral performance at school age (Karpinski et al., 2008; Touchette et al., 2007). Developmental changes in objectively assessed sleep have further been shown to relate differentially to EF components, with sleep duration and sleep efficiency showing distinct associations with working memory and inhibitory control, respectively (Bernier et al., 2021).

However, some studies on the relationship between sleep and EFs during the preschool period have yielded inconsistent results, with observed associations differing across EF domains, sleep indicators (e.g., duration versus quality), and analytic approaches, and often being small in magnitude or limited to specific subgroups (Bernier et al., 2021; Mäkelä et al., 2020; Morales-Muñoz et al., 2021; Nieto et al., 2022; Sun et al., 2022; Woods et al., 2023). Based on this heterogeneity, additional individual differences should be included in studies of the interaction between sleep quantity and quality and early developmental characteristics relevant to EF development (Nelson et al., 2020). Cross-sectional and longitudinal evidence further indicates that sleep characteristics are associated with both motor and cognitive development in early childhood, situating sleep within a broader developmental context linking these domains (Zhang et al., 2022; Liang et al., 2022). Such an approach allows for the possibility that sleep functions as a contextual moderator of an established developmental pathway, shaping the conditions under which early motor competence is more or less likely to be translated into later executive functioning through general cognitive ability. Sadeh proposed that when children have adequate rest, they can better utilize their own strengths to learn from the environment and thereby promote their own development (Sadeh, 2007). From this perspective, early motor competence may represent a developmental resource whose translation into later EF outcomes depends on the child’s sleep context. Consistent with this idea, adequate sleep during early childhood promotes synergy between development processes, leading to enhanced social–emotional functioning in young children (Schumacher et al., 2017). Further, children with high effortful control performed better on executive functioning tasks, but only if they had high sleep quality (Philbrook et al., 2022). However, both studies were cross-sectional, precluding establishment of causal relationships. Our objective here is to investigate the association between motor competence in infancy and EFs development, as well as to examine the moderating effect of sleep using longitudinal measures.

### 1.4. The Present Study

Our current prospective study aimed to expand on previous research by confirming the correlations among motor competence, general cognitive ability, and EFs in young children using an independent sample from our previous study (Wu et al., 2017). To address prior limitations, we assessed motor competence and multiple cognitive domains, including cognitive flexibility, across multiple developmental time points, allowing for a longitudinal test of mediation in early childhood. To determine whether sleep quantity and quality moderate the relationship between motor competence and EFs, we stratified children according to the recommended cutoff for good and bad sleepers of the National Health and Family Planning Commission in China (NHFPCC, 2017). Our hypothesis is that children with higher motor competence and general cognitive ability will perform better on EF tasks. Moreover, we anticipated that the relationships of motor competence and general cognitive ability with EFs would be strengthened by better sleep quantity and quality but weakened by insufficient sleep quantity and poor sleep quality.

## 2. Methods

### 2.1. Participants and Procedures

**Figure 1:**
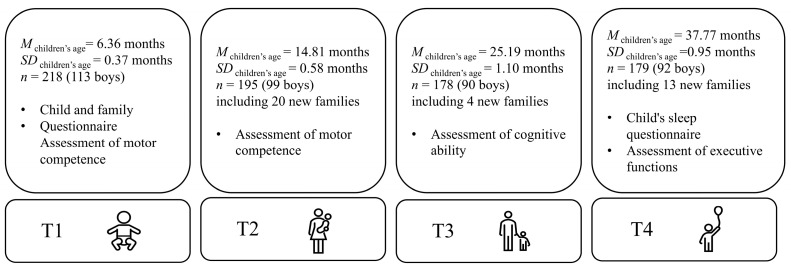
Overview of participants and their involvement across four developmental time points. Mean ages (in months), standard deviations, sample sizes, and primary assessments conducted at each wave are shown. T1 (approximately 6 months) and T2 (approximately 14 months) involved assessments of motor competence; T3 (approximately 25 months) involved assessment of general cognitive ability; and T4 (approximately 38 months) involved assessments of sleep characteristics and executive functions.

The current study enrolled 255 children (133 boys and 122 girls) and their families, all from Beijing, China. Preterm births (birth at <37 weeks) and children with birth defects that may affect neurodevelopmental outcomes were excluded. The demographics of the study population are summarized in Table A1. Ethics approval was granted by the Clinical Research Ethics Committee of Peking University First Hospital; Ref. No. 2015[871]. Parents provided informed consent on behalf of their infants. At the end of each visit, families received a gift. Children were tested from 6 months old (i.e., T1; June to December 2015) to three and a half years (i.e., T4; December 2017 to May 2018). Figure 1 presents the data collection procedures and the children’s precise ages at each time point.

Little’s Missing Completely at Random (MCAR) test indicated that our data followed the expected distribution (Little, 1988) [*χ*^2^(225) = 242.17, *p* = 0.21]. Full information maximum likelihood (FIML) was utilized to estimate the effects of missing data on the proposed models (Muthén & Muthén, 2017). This approach is ideal for managing missing data as it provides unbiased parameter estimates that surpass those obtained using other methods (Enders & Bandalos, 2001). Multiple analyses were conducted for detection of variables related to missing individual EF results. The outcomes revealed that children were more likely to have missing values if their mothers had lower educational background [*t*(206) = 2.64, *p* < 0.01] or their fathers had lower incomes [*t*(196) = 2.03, *p* < 0.05].

### 2.2. Measurements

#### 2.2.1. Motor Competence and Cognitive Ability

Motor competence was evaluated during infancy (T1 and T2) using the motor scale of the Bayley Scales of Infant and Toddler Development, Third Edition (BSID III; Bayley, 2006), while cognitive ability was assessed during toddlerhood (T3) using the cognitive scale of the same instrument. This individually administered and standardized instrument evaluates the overall motor and cognitive development of children between the ages of 1 and 42 months. The BSID III motor scale includes items grouped into two subtests, fine motor and gross motor. Scaled scores with a mean of 10 ± 3 were used in this study. Average Cronbach’s alpha coefficients were from 0.74 to 0.80. The intraclass correlation coefficients between the scores from different raters were higher than 0.95 based on 10% of the sample.

#### 2.2.2. Executive Functions

The EF tasks at age 3 (T4) were chosen in accordance with Carlson’s (2005) recommendations to measure variations in working memory, inhibition, and cognitive flexibility. The self-ordered pointing task was used to measure working memory (Hongwanishkul et al., 2005). The test had eight levels with two sections each (A and B) and an equal number of images. For example, level one had three pages with three distinct items per page. The child had to identify the items, which were in different locations. The difficulty of the levels increased, with level eight having 10 pages with 10 items each. Children first took a two-page practice quiz with two items in Version A. If they failed, they would move to Version B. Passing the practice test allowed them to take the formal test, which followed the same procedures. The score reflected the child’s ability to identify the items correctly.

The day/night Stroop task was used to assess the child’s ability to inhibit a natural response in favor of a conflicting one (Diamond et al., 2002). Specifically, the children were instructed to say “day” when they saw the moon and “night” when they saw the sun. After two successful practice trials, children were given 16 additional trials of cards to label in a predetermined order (ABBA—BAAB—ABBA—BAAB). Scores were calculated based on the total number of correct responses, with a maximum possible score of 16 points.

The Dimensional Change Card Sort (DCCS) task was used to assess cognitive flexibility (Zelazo, 2006). DCCS requires that the child sort cards; there are three rounds, and rules change for them. First, the classification must be performed based on the color of the picture, then the shape (switch), and the last round combines contradictory rules: the classification should be based on the color of the shape, depending on the presence of a frame in the picture (post switch). Participants were scored based on the trial level passed, with a score of 1 indicating a preswitch pass (five out of six cards correctly sorted), a score of 2 indicating a switch trial pass (five out of six cards correctly sorted), and a score of 3 indicating a post switch pass on the frame condition (10 out of 12 cards correctly sorted).

#### 2.2.3. Sleep Quality and Quantity

All mothers completed the validated Chinese version of the Children’s Sleep Habit Questionnaire (CSHQ; Li et al., 2007; Owens et al., 2000), which contains 33 items assessing sleep patterns and problems. The CSHQ is a reliable tool for detecting sleep problems in toddlers and preschool-aged children (Goodlin-Jones et al., 2008). In the present study, the Cronbach’s alpha coefficient for the total sleep problems scale was 0.68, which is considered acceptable for research with young children, given the heterogeneity of sleep behaviors and developmental variability in early childhood (e.g., Owens et al., 2000; Goodlin-Jones et al., 2008).

To classify sleep quality, we applied a cutoff score of 54 on the CSHQ total score, which has been deemed appropriate for younger children aged 2–5 years old (Reynolds et al., 2019), rather than the commonly used score of 41 for preschool-aged children. A dummy code of 0 was assigned to indicate a score less than 54 (high sleep quality), while a dummy code of 1 was assigned to indicate a score greater than or equal to 54 (sleep problems). Based on this cutoff, 162 children were classified as having good sleep quality, whereas 22 children were classified as having poor sleep quality.

We gathered information about the average amount of sleep by asking parents the following four questions: “What time does your child typically go to bed during the week?”, “What time does your child typically go to bed on the weekends or vacations?”, “What time does your child usually wake up on weekday mornings?”, and “What time does your child usually wake up on weekends or vacation mornings?”. To calculate the total sleep time (TST), we determined the duration between the child’s bedtime and wake-up time on both weekdays and weekends or vacations. The mean sleep duration was then calculated using the formula TST = (TST_weekday_ × 5 + TST_weekend_ × 2)/7. This weighted average was used to capture children’s habitual weekly sleep duration, consistent with prior preschool sleep research (e.g., Petit et al., 2007) and developmental accounts of sleep as a foundational condition for functioning (Sadeh, 2007). Sleep quantity was classified using a cutoff of 10 h per night, reflecting the lower bound of recommended sleep duration for preschool-aged children, informed by pediatric sleep guidelines (Paruthi et al., 2016) and national sleep hygiene recommendations (NHFPCC, 2017). Children sleeping ≥ 10 h were coded as having sufficient sleep (0), whereas those sleeping < 10 h were coded as having insufficient sleep quantity (1). Using this criterion, 154 children were classified as having sufficient sleep duration, whereas 25 children were classified as having insufficient sleep duration.

#### 2.2.4. Covariates

Gender and family socioeconomic status (SES) were included as covariates in all relevant analyses, as both are correlated with EFs (Lawson et al., 2018). To determine family SES, principal component analysis (PCA) was conducted on four variables pertaining to the educational backgrounds and monthly incomes of both parents. The Kaiser-Meyer-Olkin measure confirmed the suitability of the sample for analysis (KMO = 0.64), and the factor loadings of these variables ranged from 0.56 to 0.81. In the present study, the family SES was represented by the factor score obtained from the PCA.

### 2.3. Statistical Analysis

We first calculated descriptive statistics and correlations among the observed variables. Next, we performed confirmatory factor analysis (CFA) and calculated correlations among latent variables of motor competence and EFs. Finally, we tested the overall proposed moderated mediation model. In this analysis, motor competence during infancy was modeled as the predictor, cognitive ability during toddlerhood as the mediator, and EFs at preschool age as the outcome. Two indicators of sleep at preschool age were included as moderators of the entire cascade from infant motor competence to preschoolers’ EFs, while gender and family SES were included as covariates.

We used Mplus 8.1 to estimate the direct and interaction effects of the moderated mediation model. Significant interaction effects were then confirmed using simple slopes. Moderation effects were visualized using simple slope plots based on factor score estimates of executive functions derived from the confirmatory factor analysis. When testing indirect effects dependent on different levels of the moderator, we utilized the Mplus code provided by Stride and colleagues (Stride et al., 2015). By converting the original PROCESS macro syntax implemented in IBM SPSS Statistics Version 29.0 (IBM Corp., Armonk, NY, USA) into Mplus Version 8.1 (Muthén & Muthén, Los Angeles, CA, USA) program language, this method allows use of the FIML treatment for missing data within the framework of PROCESS (Hayes, 2013). When examining the moderated mediation effect, PROCESS uses the nonparametric bootstrapping method, which is particularly useful when the sample size is not large (MacKinnon et al., 2004). We applied the nonparametric resampling approach with 2000 resamples drawn to derive the 95% confidence intervals (CIs) for the moderated mediation effects. Given the theory-driven focus on interaction and conditional indirect effects, model comparison indices such as AIC were not used.

## 3. Results

### 3.1. Preliminary Analysis and Descriptive Statistics

All descriptive statistics and correlations among observed variables are presented in Table 1. Multiple independent *t*-tests revealed no significant gender differences [*t*(174)_working memory_ = 1.22, *p* = 0.22; *t*(149)_inhibition_ = 0.89, *p* = 0.27; *t*(176)_cognitive flexibility_ = 0.85, *p* = 0.40]. Alternatively, working memory was significantly correlated with family SES (*r* = 0.19, *p* = 0.012), with children from higher SES families performing better on working memory tasks. Thus, only family SES was included as a covariate in the following analyses.

### 3.2. Measurement Model

**Figure 2:**
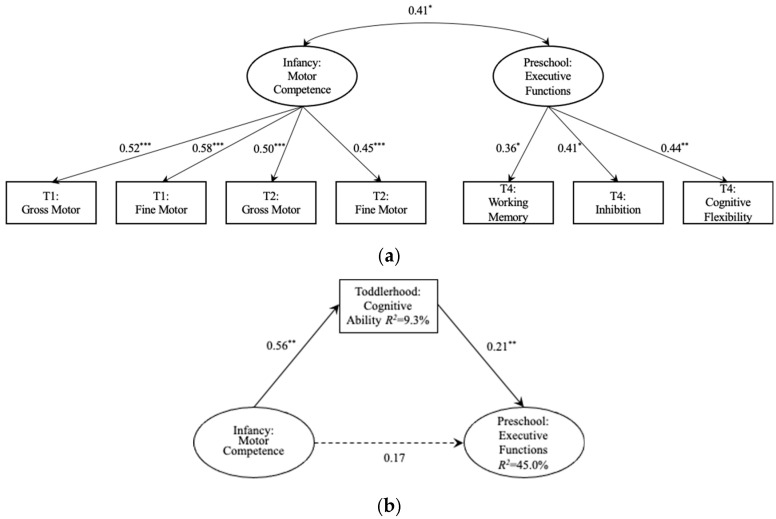
Predicting preschoolers’ executive functions from motor competence and cognitive ability. (**a**) Measurement model showing latent constructs of infant motor competence and preschool executive functions, with observed indicators including gross and fine motor skills assessed at T1 and T2, and working memory, inhibition, and cognitive flexibility assessed at T4. Standardized factor loadings are presented along each path. (**b**) Mediation model testing the indirect association between infant motor competence and preschool executive functions through toddlerhood general cognitive ability. Solid arrows indicate significant standardized paths, and the dashed arrow represents the non-significant direct path after accounting for the mediator. Values next to arrows denote standardized coefficients. Model-explained variance (*R*^2^) is shown for endogenous variables. Note: *** *p* < 0.001, ** *p* < 0.01, * *p* < 0.05.

A CFA using ML estimation was conducted to examine whether the individual indicators loaded on the constructs as expected (Figure 2a). Indeed, all standardized construct factor loadings were significant, with values ranging from 0.36 to 0.58. Additionally, the measurement model showed acceptable fit [*χ*^2^(12) = 11.65, *p* > 0.05; CFI = 1.00; TLI = 1.00; SRMR = 0.05; RMSEA = 0.00]. Motor competence during infancy was significantly correlated with EFs at preschool age (*r* = 0.41, *p* = 0.017), with infants demonstrating higher motor competence tending to perform better on future assessments of EFs.

### 3.3. Moderated Mediation Model

#### 3.3.1. Mediation Analysis

The study utilized path analysis to examine the potential indirect link between infant motor competence and EFs development through general cognitive ability in toddlerhood while considering relevant covariates. The model was a good fit for the data [*χ*^2^(17) = 15.25, *p* > 0.05; CFI = 1.00; TLI = 1.00; SRMR = 0.05; RMSEA = 0.00], with the predictors explaining 9.3% of the variance in toddlers’ general cognitive ability and 45.0% of the variance in school-age children’s EFs. As displayed in Figure 2b, regression analysis indicated that infant motor competence had a significant effect on toddlers’ general cognitive ability (β = 0.56, *p* = 0.01), which in turn had a significant effect on preschool EFs (β = 0.21, *p* = 0.01), with family SES included as a covariate in the path model. Infant motor competence was found to indirectly predict children’s EFs through general cognitive ability (Indirect effect: β = 0.12; 95% CI [0.03, 0.50]). The ratio of the indirect effect via toddlers’ general cognitive ability to the total effect of infant competence on EFs was 40.85%. These findings support the first research hypothesis of the study.

Since gender and family SES did not significantly predict EFs, they were not used as control variables in subsequent analyses. Toddlers’ general cognitive ability completely mediated the relationship, so the focus of analysis shifted to the moderating impact of sleep quality and quantity.

#### 3.3.2. Moderated Mediation Analysis

To examine how sleep quality and quantity impact preschool EFs, two models were constructed. Model 1 included the main effects of sleep quality and quantity as well as a previously observed mediation effect. The model demonstrated satisfactory fit [*χ*^2^(29) = 33.97, *p* > 0.05; CFI = 0.95, TLI = 0.92, SRMR = 0.06, RMSEA = 0.03], with a 45.5% interpretation rate. Building on Model 1, interaction item 1 (cognitive ability × sleep quantity) and interaction item 2 (cognitive ability × sleep quality) were added. Results indicated that both sleep quality and quantity had a significant moderating effect (*B*_interaction term 1_ = −0.12, 95% CI [−0.27, −0.02], and *B*_interaction term 2_ = −0.34, 95% CI [−0.90, −0.01]). Thus, hypothesis 2 was validated, demonstrating that both sleep quality and quantity moderated the second part of the mediating effect.

**Figure 3:**
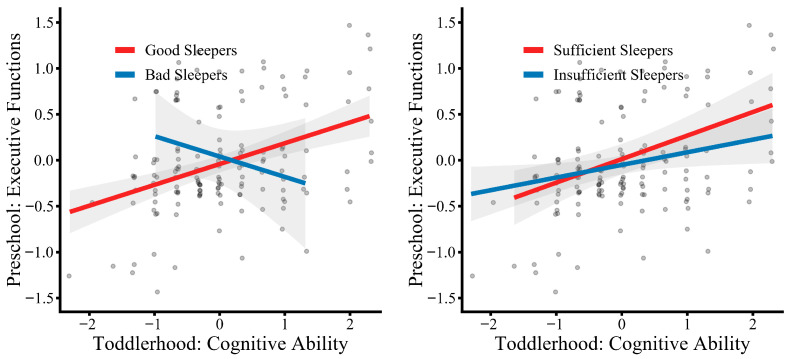
Relation between cognitive ability and executive functions in different sleep groups. Note: The (**left**) panel depicts the relation between cognitive ability and executive functions for children with good versus poor sleep quality, and the (**right**) panel depicts this relation for children with sufficient versus insufficient sleep quantity. Cognitive ability is plotted on the *x*-axis, and executive function factor scores are plotted on the *y*-axis. Points represent individual participants, and shaded bands indicate 95% confidence intervals around the fitted regression lines. Good and poor sleep quality groups were defined based on the Children’s Sleep Habits Questionnaire (CSHQ) total score cutoff, and sufficient versus insufficient sleep quantity was defined using a 10-h nightly sleep duration cutoff. Statistical significance of the simple slopes was determined based on the moderated mediation analyses and is reported in Section 3.3.2.

Additionally, simple slope tests (see Figure 3) indicated that cognitive ability in toddlerhood acted as a significant mediator (*B* = 0.09, 95% CI [0.02, 0.32]) and significantly predicted EF task performance (*B* = 0.15, 95% CI [0.03, 0.33]) for preschoolers who were good sleepers. Conversely, for preschoolers who were bad sleepers, cognitive ability in toddlerhood did not act as a significant mediator (*B* = −0.18, 95% CI [−0.77, 0.01]) nor predict performance in preschool EF tasks (*B* = −0.28, 95% CI [−0.89, 0.06]). Moreover, for preschoolers who were sufficient sleepers, cognitive ability in toddlerhood acted as a significant mediator (*B* = 0.10, 95% CI [0.01, 0.39]) and significantly predicted EF task performance (*B* = 0.16, 95% CI [0.03, 0.36]). In contrast, cognitive ability in toddlerhood did not act as a significant mediator (*B* = 0.02, 95% CI [−0.03, 0.25]) nor predict performance in preschool EF tasks (*B* = 0.04, 95% CI [−0.04, 0.29]), for insufficient sleepers. In summary, high sleep quality and adequate sleep quantity strengthened the capacity of cognitive ability in toddlerhood to predict EFs among preschoolers, while no such prediction was possible among children with insufficient sleep quantity and low sleep quality.

## 4. Discussion

The development of EFs is a multistage process (Koziol et al., 2012; Koziol & Lutz, 2013), starting with sensorimotor behaviors and progressing to basic cognitive abilities that produce mature EFs. The current study involved a three-year follow-up and used a moderated mediation model to explore the roles of general cognitive ability and sleep quality and quantity in mediating the relationship between infant motor competence and preschool EFs. Our findings underscore the importance of promoting motor competence and sleep quality and quantity for the development of EFs in preschool-age children.

Our findings confirm and expand upon those of our previous study (Wu et al., 2017). demonstrating a mediating role for cognitive ability at the toddler stage on the association between motor skills in infancy and EFs at preschool age. Additionally, our findings support the multistage EFs development hypothesis (Koziol et al., 2012; Koziol & Lutz, 2013).

Early motor skills predicted EF task performance 2 years later, consistent with previous research showing a significant association between gross and fine motor skills and EFs in preschool children (Cook et al., 2019; Maurer & Roebers, 2019). However, in previous research, only gross motor skills were associated with working memory and inhibitory control in preschool-aged children (approximately 4–6 years old) (Cook et al., 2019), while complex motor skills were more strongly correlated with EFs than simple motor skills in children aged 3–5 years (Maurer & Roebers, 2019). In contrast, a study using the Standard Assessment of Motor Coordination in Children found only a weak link between motor skills and EFs in preschool children aged 4–6 years (Veer et al., 2020). The current study used the motor subscale of the Baley III to evaluate motor skills in infancy, similar to the study by Veer et al. (2020), but found different results. This discrepancy may be explained by age at the time of task performance. The children in the current study may have already acquired a degree of automation in learning new motor skills. As a result, they may have been less reliant on executive control processes for motor performance, consistent with accounts suggesting that increasing motor automaticity reduces the need for higher-order cognitive regulation (Seger & Spiering, 2011).

This study measured children’s motor skills both before and after the ages of 6 and 14 months. Infants of this age range are in the early stages of motor skill development when motor tasks demand significant cognitive resources. For instance, crawling and walking require constant planning and adjustment based on the perception of the environment and one’s own abilities (Adolph et al., 2011; Adolph & Hoch, 2020). In hand manipulation activities, such as putting coins into a piggy bank, infants must focus on the coins, locate the slot accurately, integrate visual and motion information, and move their hands and fingers precisely over the slot to complete the action. Infants have not yet mastered these basic motor skills, and the tasks are far from automated. Thus, our findings suggest that the association between motor skills and EFs may reflect the broader cognitive demands of motor performance during early infancy, rather than specific movement types or levels of task complexity.

This study also found that cognitive ability during toddlerhood significantly predicted executive functions during the preschool years. When sleep quantity and quality were taken into account, this positive association was evident only among children with sufficient sleep quantity and high sleep quality, but not among those with insufficient or poor-quality sleep. This finding aligns with the “butterfly-type” model (Jose, 2013), which suggests that adequate and high-quality sleep can enhance cognitive development during preschool ages, but there is insufficient evidence to support the notion that lower sleep quality has a suppressive effect. The sleep quality patterns observed in the current study are similar to previous findings on effort control and cognitive ability (Berger & Scher, 2017; Philbrook et al., 2022).

This study had several limitations. First, sleep quality was an observational variable and there was no intervention to improve sleep quality among preschool children. Additionally, due to the small number of preschool children in the low sleep quality group, associations may not have reached statistical significance. Future studies may consider oversampling children with poor sleep quality and short sleep duration. Second, the EFs of preschool children were measured by one-to-one behavioral testing in the laboratory, and outcomes may have been influenced by the relationship between child and tester. Future studies should incorporate parents’ reports of their children’s daily executive functioning. Third, sleep quality and quantity estimates were based solely on reports from mothers, and differences have been found between parental reports and activity recorders (Perpétuo et al., 2020). Future studies should include data measured using objective means such as actigraphy. Finally, the families involved in the study came from a large city (Beijing) and were of relatively high socioeconomic status, so it is unclear whether the results can be generalized to typical families in other regions.

While this study did not establish a definitive causal relationship between motor skills and EFs, the strong correlations obtained have significant implications for family education practices. First, these findings underscore the importance of prioritizing infant motor skills development as suggested by the theory of embodied cognition. Caregivers are advised to familiarize themselves with general infant motor development rules and master interactive skills that promote motor development as well as other domains of development. Adequate motor skills allow children to better navigate their physical, social, and environmental surroundings, which is critical to the development of cognitive skills, including EFs (Suggate & Stoeger, 2017). Furthermore, a recent meta-analysis concluded that interventions can improve preschoolers’ sleep quality and quantity (Fangupo et al., 2021), and this study suggests that the recommended standards of China’s Sleep Hygiene Guidelines for children aged 0 to 5 years should be prioritized among infants, toddlers, and preschool-age children.

## 5. Conclusions

In summary, the development of EFs is a multistage process dependent on the acquisition of motor skills and other cognitive functions, and modulated by sleep quantity and quality. Cognitive ability during toddlerhood serves as a complete mediator between motor skills in infancy and EFs during the preschool years. The role of sleep in the development of EFs in children is not direct. Rather, the quality of preschool sleep regulates the mediating effects of cognitive ability during toddlerhood and motor skills in infancy on preschool EFs. Specifically, sufficient and high-quality sleep produces a stronger positive predictive effect of toddler-stage cognitive ability on preschool EFs, while insufficient and low-quality sleep influences the associations between cognitive ability during toddlerhood and preschool EFs.

## Figures and Tables

**Table 1:** Descriptive statistics and bivariate correlations for all study variables.

Variables	1	2	3	4	5	6	7	8	9	10	11
1. T1: Gross motor (*n* = 210)	1										
2. T1: Fine motor (*n* = 215)	0.31 **	1									
3. T2: Gross motor (*n* = 140)	0.24 **	0.28 **	1								
4. T2: Fine motor (*n* = 140)	0.14	0.29 **	0.30 **	1							
5. T3: Cognitive ability (*n* = 168)	0.24 **	0.10	0.10	0.16	1						
6. T4: Working memory (*n* = 176)	0.14	0.02	−0.01	−0.09	0.22 **	1					
7. T4: Inhibition (*n* = 151)	0.17	0.11	0.10	0.13	0.12	−0.10	1				
8. T4: Cognitive flexibility (*n* = 178)	0.20 *	0.12	−0.02	−0.02	0.34 **	0.19 *	0.15	1			
9. T4: Sleep duration (*n* = 173)	0.00	0.10	−0.11	−0.10	0.01	0.00	0.00	0.01	1		
10. T4: Sleep disturbances (*n* = 173)	0.04	−0.02	0.04	0.12	−0.09	−0.01	0.06	−0.04	−0.05	1	
11. T1: family SES (*n* = 214)	0.10	0.09	0.00	−0.13	0.05	0.19 *	0.08	0.07	0.10	−0.21 **	1
*M*	9.65	11.78	11.30	12.86	12.00	4.44	6.44	1.21	9.76	47.00	0.01
*SD*	2.43	2.69	2.78	1.97	3.04	2.52	5.77	0.55	0.85	5.72	0.99

Note: ** *p* < 0.01, * *p* < 0.05.

## Data Availability

The data that support the findings of this study are not publicly available due to ethical and privacy restrictions.

## Appendix A

**Table A1:** Family socioeconomic status of the sample.

Variable	Mother	Father
**Monthly income (Scored from 1~7)**	%	%
1. <1500 RMB	3.5	0
2. 1500~3000 RMB	3.5	0.4
3. 3000~6000 RMB	23.9	12.2
4. 6000~10,000 RMB	21.6	27.1
5. 10,000~15,000 RMB	10.6	12.5
6. 15,000~20,000 RMB	6.3	7.8
7. >20,000 RMB	4.7	17.6
Missing	25.9	22.4
**Parental education (Scored from 1~4)**		
1. Middle school or below	0.8	0.4
2. High school or vocational school degree	4.3	4.3
3. Bachelor’s degree or vocational college degree	51.8	48.6
4. Master’s degree or above	24.7	25.5
Missing	18.4	21.2
